# A retrospective study of tuberculosis prevalence and associated factors among HIV-positive key populations in Nigeria

**DOI:** 10.1371/journal.pgph.0003461

**Published:** 2024-07-12

**Authors:** Bartholomew Ochonye, Olaniyi Felix Sanni, Godwin Emmanuel, Paul Umoh, Abiye Kalaiwo, Roger Abang, Paul Amechi, Mark Ahkigbe, Shakirat Akinpelumi, Olugbemi Motilewa

**Affiliations:** 1 Research and Development Department, Heartland Alliance, Abuja, Nigeria; 2 Program Management, USAID, Abuja, Nigeria; 3 Department of Community Medicine, University of Uyo Teaching Hospital, Uyo, Akwa Ibom State, Nigeria; Chinese Academy of Medical Sciences and Peking Union Medical College, CHINA

## Abstract

HIV is a major risk factor for active Tuberculosis (TB.) This raises patients’ risk of original infection, reinfection, and TB reactivation. Providing healthcare to KPLHIV in developing countries requires TB prevalence research. This study aims to determine the prevalence of TB and HIV co-infection and associated factors among KPLHIV. This is a retrospective cross-sectional study among KP’s living with HIV enrolled on care in One Stop Shop (OSS) of Heartland Alliance Ltd/GTE across six states in Nigeria. Data were analysed using IBM SPSS version 25.0. Secondary data analysis of client’s records from the RADET files of the KPCARE 1 project from 6 states was conducted. Means with standard deviations were computed for continuous variables like age, and frequency tables were generated for categorical variables. Chi-square tests and t-tests were used for the bivariate analysis of variables. All tests were done at a 5% level of statistical significance (p = 0.05).TB prevalence was 19.1% among KP’s living with HIV, with variations observed in age groups, geographic locations, target populations, marital status, educational backgrounds, clinical characteristics, and antiretroviral therapy (ART) history. KPs aged 51 and above exhibited the highest TB prevalence (21.0%), while those aged below 20 years had the lowest (18.2%). Jigawa KPs recorded the highest TB prevalence (38.4%), and Niger had the least (13.3%). TB was more prevalent among People who inject drugs (20.3%), divorced (32.3%), and those who attained Qur’anic education (29.7%). KPs who had to restart ART exhibited the highest TB prevalence (22.0%), whereas those who experienced Interruption in treatment (IIT) reported the lowest at 10.0%. Immune-suppressed KPs (CD4 counts < 200 cells/m^3^) had a higher TB prevalence of 26.6%. TB prevalence among KPs living with HIV varies greatly, underlining the need for targeted treatments, especially for high-risk categories, to improve HIV treatment outcomes and reduce TB prevalence.

## Introduction

The co-occurrence of HIV and Tuberculosis (TB) remains a major public health issue worldwide, especially in low- and middle-income countries (LMICs) [1]. In 2019, the World Health Organization (WHO) reported that around 38 million individuals were HIV-positive, with approximately 10 million new cases of TB [2]. These significant figures highlight the critical need to tackle both epidemics, especially among vulnerable communities.

The majority of the key populations (KPs), including Men Who Have Sex with Men (MSM), Female Sex Workers (FSW), and People Who Inject Drugs (PWID), are disproportionately affected by both HIV and TB due to a range of social, economic, and structural factors, including stigma, discrimination, and limited access to healthcare services [3, 4]. These groups often exist at the margins of society, making them more susceptible to health risks and less likely to access timely and appropriate medical care.

The relationship between HIV and TB is particularly complex among KPs. HIV is a potent risk factor for the activation of latent TB, making co-infected individuals more susceptible to severe forms of TB and complicating the clinical management of both diseases [5, 6]. Among KPs, the prevalence of TB-HIV co-infection is alarmingly high, often exacerbated by behaviours such as unprotected sex and needle sharing, which increase the risk of acquiring both infections [5, 7].

The severity of HIV/TB in the general population can vary significantly depending on factors such as geographic location, access to healthcare, socioeconomic status, and prevalence rates. In regions with well-established healthcare systems and effective public health interventions, the burden of HIV/TB is generally lower due to better access to testing, treatment, and prevention services [8, 9]. However, in areas with limited healthcare infrastructure, lack of awareness, and social stigma surrounding HIV/TB, the severity can be higher [10]. Late diagnosis, inadequate treatment, and barriers to care contribute to the impact of HIV/TB on the general population [11]. Stigma and discrimination also play a role in deterring individuals from seeking testing and treatment, leading to increased transmission rates and poorer health outcomes [12].

When compared with KP, the severity of HIV/TB is a significant concern due to various factors. These populations often engage in high-risk behaviors such as unprotected sex, or having multiple sexual partners, increasing their vulnerability to HIV and TB transmission [13]. Also, factors like poverty, social exclusion, and legal barriers can limit their access to quality healthcare services, leading to undiagnosed or untreated HIV/TB infections [14]. HIV/TB coinfection is common among these populations, leading to more severe health outcomes and challenges in treatment due to drug interactions and compromised immune systems [15]. Engaging these populations in HIV/TB prevention and treatment programs can be challenging due to various factors like mobility, fear of disclosure, and distrust of healthcare systems. Prisons often have overcrowded and unsanitary conditions, increasing the risk of HIV/TB transmission among inmates who may lack access to adequate healthcare services [14].

In Sub-Saharan Africa, where the burden of HIV is highest, TB co-infection rates among KPs are particularly concerning [16]. Nigeria, one of the 30 high TB burden countries, has a significant prevalence of TB-HIV co-infections, especially among KPs [17]. The healthcare system in the country is dealing with several difficulties, such as insufficient funding, substandard infrastructure, and a scarcity of healthcare professionals, all of which worsen the situation. Managing TB-HIV co-infections is particularly problematic due to issues like drug interactions, overlapping toxic effects, and the potential for Immune Reconstitution Inflammatory Syndrome (IRIS) [18]. These complexities necessitate an integrated approach to the clinical management of co-infected individuals, particularly among KPs who may face additional barriers to accessing healthcare services.

Despite the wealth of studies on HIV and TB co-infections, there is a notable gap in the literature concerning the prevalence of TB and its associated factors among KP’s living with HIV in Nigeria. This study aims to fill this gap by conducting a retrospective analysis of KPs data from the One Stop Shop (OSS) of Heartland Alliance Ltd/GTE in Nigeria. Understanding the interplay between TB and HIV among KPs is crucial for developing targeted interventions to improve HIV treatment outcomes and reduce TB co-infection rates. The co-occurrence of HIV and TB among KPs in Nigeria presents a complex public health challenge that requires a multi-faceted approach. This research will add to the existing body of knowledge by uncovering the prevalence of TB among people with HIV in Nigeria as well as the factors linked to it. The results could help shape public health plans and actions aimed at lessening the impact of both HIV and TB on these at-risk groups.

## Methodology

### Study design

This research employed a retrospective study design to investigate the prevalence of TB (TB) and its associated factors among KP’s living with HIV in Nigeria. The study focused on six distinct groups: FSW, MSM, Prison Inmates, PWID, Sexual Partners (SP), and Transgender Individuals (TG). These groups are all participants in the Heartland Alliance LTD/GTE One Stop Shops facilities in six targeted states (Lagos, Bayelsa, Cross River, Akwa Ibom, Jigawa, and Niger) in Nigeria. Unlike studies that rely on sampling methods, this research included all HIV-positive individuals enrolled in the HA LTD/GTE OSS program regardless of their ART status. This approach yielded a comprehensive data set, facilitating an exhaustive analysis of TB prevalence and associated factors among these KPs.

### Data source

Data for this study were extracted from the HA LTD/GTE OSS databases in the six selected states, spanning three years from May 2019 to September 2022. The data was used and assessed on the 15^th^ of December, 2023. The aim was to provide a thorough understanding of TB prevalence and its associated socio-demographic and clinical factors among KP’s living with HIV.

### Data analysis

Before analysis, the datasets were rigorously cleaned using Microsoft Excel to ensure their accuracy and completeness. This involved the removal of duplicate entries, addressing missing values, and verifying data integrity. The cleaned data were then imported into IBM SPSS Statistics for further analysis.

The study utilized descriptive statistics to summarize the socio-demographic and clinical characteristics of the study population. This involved calculating frequencies and percentages for factors like gender, age, education level, employment status, marital status, and various clinical aspects. Chi-square tests examined the relationship between these variables and TB prevalence among the KPs. Logistic regression analysis was used to examine how socio-demographic and clinical factors related to TB development in HIV-positive individuals within the KP community. Odds ratios (ORs) with 95% confidence intervals were calculated, and variables with a p-value below 0.05 in the initial analysis were included in the final model. All tests were two-sided, and a p-value under 0.05 was deemed significant.

### Ethical considerations

This study was conducted under Heartland Alliance’s approved KP-CARE 1 ethical clearance for project implementation research. The Health Research Ethics Committee (HREC) assigned the number UUTH/AD/S/96/VOL.XXI/812. This was adapted to ensure stringent data protection and security compliance once HALG granted permission to use the KPs’ data. To ensure participant confidentiality and privacy, rigorous measures were taken to anonymize and securely store the collected data.

## Results

### Prevalence of TB by socio-demographic of KPs living with HIV

As shown in Table 1, the overall TB prevalence was 19.1%. The prevalence of TB is quite similar between males and females: 19.1% for males and 19.1% for females out of their respective totals of 50,015 and 63,884. KPs aged 51 and above have the highest TB prevalence at 21.0%, followed by those aged 41–50 at 20.6%. The lowest TB prevalence is among those under 20, at 18.2%. Among the states, Jigawa has a notably high TB prevalence of 38.4%, followed by Cross Rivers at 28.2%. Niger has the lowest TB prevalence of 13.3%.

**Table 1:** Prevalence of TB by socio-demographic of KPs living with HIV (N = 113899).

	Variable	Frequency (n = 113899)	Percentage (%)	TB Prevalence % (n = 21794)
**Gender**	Male	50015	43.9	9576 (19.1)
	Female	63884	56.1	12218 (19.1)
**Age Category**	≤ 20	1138	1.0	207 (18.2)
	21–30	42384	37.2	7697 (18.6)
	31–40	46386	40.7	8930 (19.3)
	41–50	19574	17.2	4031 (20.6)
	51 +	4417	3.9	929 (21.0)
**State**	Akwa Ibom	33509	29.4	5054 (15.1)
	Bayelsa	10074	8.8	2048 (20.3)
	Cross Rivers	30108	26.4	8491 (28.2)
	Jigawa	1701	1.5	654 (38.4)
	Lagos	28909	25.4	4269 (14.8)
	Niger	9598	8.4	1278 (13.3)
**Target Group**	FSW	52673	46.2	9978 (18.9)
	MSM	31505	27.7	5964 (18.9)
	Prison	821	0.7	158 (19.2)
	PWID	16020	14.1	3260 (20.3)
	SP	10298	9.0	1980 (19.2)
	TG	2582	2.3	454 (17.6)
**Marital status**	Single	98519	86.5	18179 (18.5)
	Married	14282	12.5	3356 (23.5)
	Separated	351	0.3	92 (26.2)
	Widowed	524	0.5	95 (18.1)
	Divorced	223	0.2	72 (32.3)
**Education**	Primary	10127	8.9	2075 (20.5)
	Quaranic	231	0.2	69 (29.9)
	Junior Secondary	2510	2.2	561 (22.4)
	Senior Secondary	73178	64.2	13355 (18.3)
	Post-secondary	27853	24.5	5734 (20.6)
**Occupation**	Unemployed	40574	35.6	7921 (19.5)
	Student	15024	13.2	2884 (19.2)
	Employed	58201	51.1	10960 (18.8)
	Retired	100	0.1	29 (29.0)

Regarding the target groups, People who inject drugs (PWID) have a TB prevalence of 20.3%, the highest among the groups, whereas the Transgender group (TG) has the lowest at 17.6%. Marital status also seems to affect TB prevalence, with divorced individuals showing the highest prevalence at 32.3% and singles having the lowest at 18.5%. KPs with Qur’anic education have the highest TB prevalence of 29.9%, while those with senior secondary education have the lowest at 18.3%. Retired individuals have the highest TB prevalence at 29.0%, and employed individuals have the lowest at 18.8%.

### Prevalence of TB by clinical characteristics of KPs living with HIV

As shown in Table 2, KPs who had to restart ART have the highest prevalence of TB at 22.0%, followed closely by those who were transferred between ART programs with a prevalence of 21.6%. On the other hand, individuals who experienced Interruption in Treatment (IIT) have the lowest TB prevalence at 10.0%, and those who have stopped treatment have a prevalence of 13.0%. KPs weighing less than or equal to 50 Kg show a higher TB prevalence of 22.7% compared to those weighing more than 50 Kg, who have a prevalence of 18.6%. Regarding CD4 count, those with a count of less than 200 cells/m3 show a markedly higher TB prevalence of 26.6%, in contrast to those with a count of 200 cells/m3 or more, who show a prevalence of 14.1%. The clinic stage after diagnosis also provides some insights, although most of the sample falls under Stage I with a TB prevalence of 19.1%. Stage III has a slightly higher TB prevalence at 21.2%, while Stage IV has the lowest at 16.7%.

**Table 2:** Prevalence of TB by clinical characteristics of HIV- positive KPs (N = 114330).

	Variable	Frequency (n = 113899)	Percentage (%)	TB Prevalence % (n = 21794)
**ART status**	ART Restart	865	0.8	190 (22.0)
	ART Start	110108	96.7	21126 (19.2)
	ART Transferred	268	0.2	58 (21.6)
	ART Transferred out	1564	1.4	270 (17.3)
	Died (Confirmed)	281	0.2	55 (19.6)
	IIT	359	0.3	36 (10.0)
	Stopped Treatment	454	0.4	59 (13.0)
**Weight Category**	≤ 50Kg	15557	13.7	3533 (22.7)
	> 50 Kg	98193	86.3	18231 (18.6)
**CD4 (cell/m** ^**3**^ **) (n = 2037)**	< 200 cell/m^3^	158	7.8	42 (26.6)
	≥200 cell/m^3^	1879	92.2	265 (14.1)
**Clinic stage**	Stage I	113716	99.8	21763 (19.1)
	Stage II	132	0.1	21 (15.9)
	Stage III	33	0.0	7 (21.2)
	Stage IV	18	0.0	3 (16.7)

### Association between socio-demographic and clinical characteristics with the development of TB in KP’s living with HIV

As shown in Table 3, the prevalence of TB among KP’s living with HIV varies by socio-demographic and clinical characteristics. Among different age groups, the prevalence of TB is highest among those aged 51 (21.0%) and lowest among those aged below 20 (18.2%). However, these associations were not statistically significant in the adjusted model. TB prevalence was highest in Jigawa (38.4%) and lowest in Niger (13.3%). The odds of developing TB were significantly higher in Jigawa, with an adjusted OR of 4.10 (P < 0.001) and in Cross Rivers, with an adjusted OR of 2.54 (P < 0.001). TB prevalence was comparatively higher among PWID at 20.3%. The odds of developing TB in this group were significantly low, with an adjusted OR of 0.95 (P = 0.383).

**Table 3:** Association between socio-demographic and clinical characteristics with the development of TB in KP’s living with HIV.

Variable	TB Prevalence (%)	Unadjusted		Adjusted	
		OR (95% CL)	P value	OR (95% CL)	P value
**Age category**					
≤ 20	207 (18.2)	Ref	-		
21–30	7697 (18.6)	0.998 (0.857–1.163)	0.980	1.00 (0.86–1.74)	0.945
31–40	8930 (19.3)	1.072 (0.921–1.249)	0.369	1.05 (0.90–1.22)	0.532
41–50	4031 (20.6)	1.166 (0.999–1.361)	0.051	1.13 (0.97–1.33)	0.112
51 +	929 (21.0)	1.198 (1.014–1.416)	0.034	3.35 (0.98–1.39)	0.067
**State**					
Akwa Ibom	5054 (15.1)	1.156 (1.082–1.235)	<0.001*	1.14 (1.06–1.22)	<0.001*
Bayelsa	2048 (20.3)	1.661 (1.539–1.793)	<0.001*	1.68 (1.55–1.82)	<0.001*
Cross Rivers	8491 (28.2)	2.557 (2.399–2.726)	<0.001*	2.54 (2.37–2.72)	<0.001*
Jigawa	654 (38.4)	4.067 (3.628–4.558)	<0.001*	4.10 (3.64–4.62)	<0.001*
Lagos	4269 (14.8)	1.128 (1.055–1.206)	<0.001*	1.11 (1.04–1.19)	0.003
Niger	1278 (13.3)	Ref	-	Ref	-
**Target Group**					
FSW	9978 (18.9)	1.095 (0.988–1.215)	0.085	1.00 (0.89–1.10)	0.960
MSM	5964 (18.9)	1.094 (0.985–1.216)	0.092	1.04 (0.93–1.15)	0.534
Prison	158 (19.2)	1.117 (0.914–1.366)	0.280	1.17 (0.95–1.43)	0.139
PWID	3260 (20.3)	1.198 (1.075–1.335)	0.001*	0.95 (0.85–1.06)	0.383
SP	1980 (19.2)	1.116 (0.997–1.249)	0.057	0.95 (0.84–1.07)	0.382
TG	454 (17.6)	Ref	-	Ref	-
**Marital status**					
Single	18179 (18.5)	Ref	-	Ref	-
Married	3356 (23.5)	1.357 (1.302–1.416)	<0.001*	1.17 (1.17–1.22)	<0.001*
Separated	92 (26.2)	1.570 (1.237–1.992)	<0.001*	1.27 (0.99–1.62)	0.060
Widowed	95 (18.1)	0.979 (0.783–1.223)	0.849	0.89 (0.71–1.12)	0.334
Divorced	72 (32.3)	2.107 (1.591–2.791)	<0.001*	2.32 (1.74–3.10)	<0.001*
**Education**					
Primary	2075 (20.5)	Ref	-	Ref	-
Qur’anic	69 (29.9)	1.653 (1.653–2.200)	0.001*	1.04 (0.77–1.41)	0.792
Junior Secondary	561 (22.4)	1.117 (1.005–1.241)	0.040*	1.05 (0.94–1.17)	0.350
Senior Secondary	13355 (18.3)	0.866 (0.823–0.912)	<0.001*	1.04 (0.98–1.10)	0.148
Post-secondary	5734 (20.6)	1.006 (0.951–1.064)	0.836	0.98 (0.92–1.04)	0.542
**Occupation**					
Unemployed	7921 (19.5)	Ref	-	Ref	-
Student	2884 (19.2)	0.979 (0.934–1.027)	0.388	0.97 (0.92–1.02)	0.340
Employed	10960 (18.8)	0.958 (0.926–0.988)	0.007*	0.91 (0.88–0.95)	<0.001*
Retired	29 (29.0)	1.684 (1.092–2.595)	0.018*	1.03 (0.66–1.60)	0.888
**Current Status**					
ART Restart	190 (22.0)	1.884 (1.372–2.588)	<0.001*	1.92 (1.40–2.65)	<0.001*
ART Start	21126 (19.2)	1.589 (1.209–2.090)	0.001*	1.85 (1.40–2.44)	<0.001*
ART Transferred	58 (21.6)	1.849 (1.240–2.756)	0.003*	1.80 (1.20–2.71)	0.004*
ART Transferred out	270 (17.3)	1.397 (1.031–1.892)	0.031*	1.52 (1.12–2.07)	0.007*
Died (Confirmed)	55 (19.6)	1.629 (1.090–2.436)	0.017*	1.60 (1.06–2.39)	0.025*
IIT	36 (10.0)	0.746 (0.481–1.158)	0.192	0.98 (0.64–1.56)	0.982
Stopped Treatment	59 (13.0)	Ref	-	Ref	-
**Weight Category**					
≤ 50Kg	3533 (22.7)	1.289 (1.237–1.342)	<0.001*	1.43 (0.96–2.11)	0.078
> 50 Kg	18231 (18.6)	Ref	-	Ref	-
**CD4 (cell/m** ^**3**^ **)**					
< 200 cell/m^3^	42 (26.6)	2.205 (1.514–3.212)	<0.001*	1.40 (0.93–2.12)	0.110
≥200 cell/m^3^	265 (14.1)	Ref	-	Ref	-

Divorced KPs had the highest TB prevalence at 32.3%. They also had significantly higher odds of developing TB with an adjusted OR of 2.32 (P < 0.001). Education-wise, those with Qur’anic education had the highest prevalence at 29.9%, but the association was not statistically significant in the adjusted model. Retired individuals had the highest TB prevalence (29.0%), but the association was insignificant in the adjusted model. Those who were in the "ART Restart" category had a TB prevalence of 22.0% and significantly higher odds of developing TB with an adjusted OR of 1.88 (P < 0.001). KPs weighing less than or equal to 50 Kg had a higher TB prevalence of 22.7%, but the association was not statistically significant in the adjusted model. Finally, KPs with a CD4 count of fewer than 200 cells/m3 had a TB prevalence of 26.6%, which was not statistically significant in the adjusted model.

## Discussion

This study examines the prevalence of HIV-TB co-infection among KPs in Nigeria. Rajian et al. state that dealing with both HIV and TB infections concurrently poses a major difficulty to the efficacy of ART in HIV treatment because over 60% of individuals infected with HIV also acquire TB [19]. Patients diagnosed with TB warrant particular attention because of the complexities associated with managing HIV and TB concurrently. This is primarily due to the potential medication interactions between rifampicin and non-nucleoside reverse transcriptase inhibitors (NNRTIs) and protease inhibitors (PIs), as well as challenges related to pill load, adherence, and drug toxicity [19].

The study reports an overall TB prevalence of 19.1% among KP’s living with HIV. This prevalence rate is noteworthy as it indicates a significant burden of TB within this population. This prevalence rate is consistent with previous research by Dememew et al., who reported a similar TB prevalence of 19.5% among KP’s living with HIV in Ethiopia [20]. The high prevalence of TB among KP’s living with HIV raises concerns about the effectiveness of existing prevention and control measures. It stresses how crucial it is to have specific interventions aimed at tackling this co-infection and improving overall health outcomes for KPs. The study found similar prevalence rates among males and females, suggesting that both genders face a relatively equal risk of TB co-infection within this population. This suggests that TB infection does not exhibit a significant gender bias within KP’s living with HIV. This observation contrasts with the findings of [21], who reported gender-based differences in TB prevalence among HIV-positive populations. They found a higher prevalence among females compared to males.

The findings show that TB prevalence varies significantly by age group, with the highest prevalence among KPs aged 51 and above (21.0%). The age-related variation in TB prevalence might be attributed to several factors. Older individuals may have a longer history of HIV infection, increasing their exposure to TB. Additionally, ageing weakens the immune system, making older KPs more susceptible to TB. This is corroborated by previous research by [22, 23], that has identified age as a risk factor for TB co-infection in HIV-positive individuals. In their research, they discovered that individuals over 40 years old had 2.7 times higher odds of having TB/HIV co-infection compared to those under 25 years old. This higher risk among older patients may stem from their increased vulnerability to both new and reactivated M. TB infections, as older age often correlates with a higher reservoir for M. TB [24, 25]. Managing TB in this age group is complex due to their tendency toward chronic health conditions and negative reactions to medications, leading to challenges in treatment control and higher mortality rates linked to declining immunity [26–28]. Ageing typically brings about physiological changes in the lungs, resulting in persistent low-grade inflammation, decreased lung function, and weakened immune responses, all of which contribute to the heightened susceptibility of older individuals to lung diseases and respiratory infections [29, 30]. Furthermore, elderly individuals exhibit decreased lung tissue-repair capabilities compared to younger counterparts [31].

Moreover, older individuals may face heightened exposure to environmental risk factors like indoor air pollution, overcrowded living conditions, and limited healthcare access, all of which can increase the transmission of TB and contribute to its higher prevalence among ageing populations [32, 33]. Tailored interventions for different age groups within KP’s living with HIV are needed. Older individuals should receive special attention and more rigorous screening for TB co-infection.

Also, the findings highlight substantial regional differences in TB prevalence, with Jigawa and Cross Rivers states reporting notably high rates of 38.4% and 28.2%, respectively. In comparison, Niger state has the lowest prevalence at 13.3%. Regional disparities may be influenced by various factors, including differences in healthcare infrastructure, access to care, and prevalence of both HIV and TB within the general population [34]. These regional disparities may also be due to variations in socio-economic status and living conditions. The findings emphasise the need for targeted interventions in high-prevalence regions like Jigawa and Cross Rivers. These regions require enhanced healthcare infrastructure, improved access to diagnostic services, and comprehensive prevention strategies.

Furthermore, the findings revealed several interesting patterns related to TB prevalence. Notably, PWIDs have the highest TB prevalence at 20.3% among the Target groups, divorced individuals show the highest prevalence at 32.2%, and KPs with Qur’anic education have the highest TB prevalence at 29.7%. The high TB prevalence among PWID could be attributed to crowded spaces when injecting drugs, with the susceptibility increasing for those living in low-income areas with limited access to health care [35]. Injecting drugs makes PWID more vulnerable to M. TB infection, especially in socio-economic circumstances with poor healthcare access, congested housing conditions, and the usage of highly populated injection sites. Poor community adherence to TB treatment adds to this risk, highlighting the intricate interaction of factors linked with illicit drug use [35, 36]. Additionally, health illiteracy and poor knowledge/awareness can also indeed contribute significantly to the high prevalence of TB (TB) among PWID [15]. Early exposure in youth often results in poor school attendance or even dropping out, which in turn heightens the likelihood of drug use, leading to further disengagement from education. Consequently, this lack of education contributes to limited awareness of associated risks or the importance of seeking healthcare [37]. Research confirms that individuals with no or average schooling face nearly double the risk of TB infection compared to those with higher levels of education, highlighting the correlation between education levels and health outcomes [15]. Moreover, higher education levels not only correlate with increased knowledge about health risks but also with reduced delays in diagnosing TB [15].

Divorced individuals may face additional stressors that compromise their immune systems, and KPs with Qur’anic education may have limited access to healthcare and TB awareness. These findings align with the study by [38] on TB and HIV co-infection, which reported varying prevalence rates among target groups and marital statuses. Also, the high prevalence of TB incidents among the divorce individuals in this study was similar when compared to a case-control study in Southwest Ethiopia by [39] in which 32.1% of divorce individuals had active TB. This study’s findings were contrary to studies by [40] in Nigeria and [41] in Zambia. Their study found a high prevalence of TB among married individuals. The possible reasons for a high prevalence of TB among Separated and divorced individuals could stern from the fact that this population are more susceptible to both physical and mental health issues when compared to those who are married [42]. Those who have gone through separation or divorce in the past have higher levels of depressive symptoms and feelings of loneliness compared to their married counterparts [43]. These individuals also exhibited poorer immune function and experienced more recent illnesses. This weakened immune system can make them more susceptible to TB [43]. Furthermore, men whose wives initiated the separation, indicating less control over the situation, tended to experience greater distress and poorer overall health compared to those who initiated the separation themselves [43]. These findings underscore the importance of tailored interventions for specific subgroups within KPs. HIV patients who inject drugs, divorced individuals, and those with limited formal education need targeted healthcare and support services to mitigate their higher risk of TB co-infection. In addition, the study indicates that retired individuals have the highest TB prevalence at 29.0%, while employed individuals have the lowest at 18.9%. This variation may be due to differences in healthcare access and lifestyle factors. Retired individuals may have limited access to healthcare, while those employed may have better access to preventive measures and healthcare services. Retired KPs require special attention and increased efforts to improve their healthcare access and TB prevention strategies. These findings corroborate the findings of [44, 45], who also observed an association between education, employment, and TB prevalence among HIV-positive individuals.

One striking observation in the findings is the high prevalence of TB among KPs who had to restart ART (21.9%) and those who were transferred between ART programs (21.6%). This finding may be attributed to several factors. Firstly, individuals restarting ART or transferring between programs may have experienced interruptions in their HIV treatment, which makes them more susceptible to opportunistic infections, including TB. In previous studies, disruptions in treatment adherence have been associated with increased TB risk [46, 47]. Secondly, these KPs might have more advanced HIV disease progression, lower CD4 counts, or poorer overall health, which could elevate their susceptibility to TB co-infection [48, 49]. Also, Several research studies have pointed out that interruptions in ART can result in a compromised immune system, increasing the vulnerability of individuals to opportunistic infections like TB [50–52].

Additionally, per the findings, it is noteworthy that individuals who experienced interruptions in treatment (IIT) have the lowest TB prevalence. This finding could be related to the fact that IIT might have been relatively short, and patients quickly resumed treatment, minimising the negative impact on their immune system. Shorter treatment interruptions are less likely to lead to significant CD4 count declines [53]. This finding might seem counterintuitive, as one would expect that discontinuing treatment would increase susceptibility to TB. PEPFAR’s 2020 definition of IIT as the absence of clinical contact for a minimum of 28 days after the last clinical appointment or anticipated clinic visit. This definition aligns with WHO’s concept of loss to follow-up (LTFU), differing primarily in the duration of no clinical contact, set at 90 days by WHO [54]. Research indicates that treatment interruptions, whether planned or unplanned, elevate the risk of opportunistic infections and mortality [55, 56]. Notably, the most significant viral load increase and associated CD4 decline occur within the initial two months of such interruptions [57]. Concerns related to drug resistance and heightened mortality rates parallel those of sub-optimal adherence during these interruptions [56, 58, 59]. The relatively lower TB prevalence of 10.0% among individuals who experienced IIT is intriguing and suggests that there may be factors contributing to this group’s reduced risk of TB compared to others in this study. One possible explanation could be the provision of targeted support and interventions during treatment interruptions. For instance, programs that implement directly observed therapy (DOT) or provide enhanced adherence support during periods of treatment interruption can help ensure that individuals continue to receive their medications and adhere to their treatment regimens [60, 61]. This approach has been shown to improve treatment outcomes and reduce the risk of opportunistic infections like TB among people living with HIV. Additionally, efforts to address the underlying reasons for treatment interruptions, such as addressing medication side effects, mental health concerns, or social determinants of health, may also play a role in reducing TB prevalence. For example, offering counseling, mental health support services, or addressing issues related to housing and food security can help individuals better manage their HIV treatment and overall health [62, 63].

This study found an association between TB prevalence and weight, CD4 count, and clinical stage. KPs weighing less than or equal to 50 kg exhibit a higher TB prevalence (22.7%) than those weighing more than 50 kg (18.6%). This difference may be because lower body weight often indicates poorer nutritional status, which can weaken the immune system’s ability to fend off TB [64, 65]. The study by Nagpal et al. also reiterates that malnutrition and lower body weight are known risk factors for TB [66]. Malnutrition can compromise the immune system, increasing susceptibility to infections like tuberculosis (TB) [66]. A low body mass index (BMI) often signifies inadequate nutrition, further raising the risk of TB and other opportunistic infections in individuals with HIV/AIDS. Multiple studies have shown a higher TB occurrence in those with low BMI or underweight conditions [67–69]. Malnutrition not only hampers immune function but also diminishes the body’s capacity to combat infections and can impede TB treatment effectiveness [69]. Additionally, malnourished individuals may experience delayed recovery and increased mortality rates when co-infected with TB and HIV. The higher TB prevalence among KPs weighing less than or equal to 50 kg suggests a potential link between nutritional status and TB susceptibility. Addressing malnutrition and improving nutritional status should be integrated into comprehensive TB and HIV/AIDS care programs. Nutritional support, including supplementation and counseling, can play a crucial role in enhancing immune function, improving treatment outcomes, and reducing the burden of TB among vulnerable populations [70–73].

Moreover, KPs with CD4 counts below 200 cells/m3 have a significantly higher TB prevalence (26.6%) than those with CD4 counts of 200 cells/m3 or more (14.1%). This aligns with established evidence that a weakened immune system, as indicated by low CD4 counts, is a major risk factor for TB co-infection among HIV-positive individuals [74]. Previous research has established that malnutrition and low CD4 counts are risk factors for TB in HIV-positive individuals [75–77]. Individuals with lower CD4 counts are more immunocompromised and are at a greater risk of TB [76]. The findings show that in terms of clinical stage, while most of the sample falls under Stage I with a TB prevalence of 19.2%, Stage III exhibits a slightly higher TB prevalence at 21.2%, and Stage IV has the lowest prevalence at 16.7%. This discrepancy could be attributed to the advanced immune suppression and weakened health often seen in Stage IV. This may decrease TB prevalence due to higher mortality rates or more focused clinical management [78]. Studies have indicated that advanced HIV increases TB risk, especially in Stage III [18, 79]. Aaron et al. also reported that Individuals in more advanced HIV clinical stages (Stage III) are likely to have weakened immune systems and a higher susceptibility to TB [80].

The study observed variations in TB prevalence among different age groups, with the highest prevalence among individuals aged 51 and above. However, these associations were not statistically significant after adjusting for other variables. This finding suggests that while older age may be a risk factor for TB, it is likely influenced by other factors such as immune status, duration of HIV infection, and access to healthcare [81]. The lack of statistical significance in age groups may be due to a small sample size or a more complex relationship between age and TB in this population. This suggests that age alone may not strongly predict TB among KP’s living with HIV. It could be essential to explore other contributing factors, such as immune status or healthcare access, in future research. Several studies have indicated that older HIV-positive individuals often have compromised immune systems, making them more susceptible to TB co-infection [82–84].

The findings showed that TB prevalence was significantly higher in Jigawa and Cross Rivers states. The adjusted odds ratios (OR) suggest that individuals in these regions are at a greater risk of developing TB. This could be attributed to regional variations in healthcare infrastructure, TB control programs, and socio-economic factors. Regional disparities in TB prevalence might be attributed to differences in healthcare infrastructure, awareness, and access to services. High prevalence in Jigawa could also be influenced by environmental factors or local epidemiology. These findings emphasise the importance of region-specific interventions and resource allocation to address TB co-infection among KP’s living with HIV. It may also suggest the need for targeted public health campaigns in high-prevalence areas.

Research has shown that TB incidence can vary significantly across different geographic regions due to differences in healthcare access and socio-economic status [85–87]. The study found a significantly higher frequency of TB among PWID who are also HIV-positive. This raises serious concerns about the increased likelihood of co-infection in this cohort. PWID face unique problems, such as limited and delayed healthcare access, which may extend their infectious period before seeking treatment. As a result, prompt testing and treatment for M. TB infection in this population is critical for lowering incidence rates and preventing further TB transmission [88]. The study found that divorced KPs exhibited the highest TB prevalence and significantly higher odds of developing TB. Divorce’s social and economic implications may contribute to this vulnerability, as divorced individuals may face increased stress [43]. Divorced individuals may face social and economic stressors that may likely increase their susceptibility to TB. These stressors might include unstable housing and limited access to healthcare [43, 89]. Identifying divorced KPs as a high-risk group suggests the importance of targeted interventions and support services for this population, addressing medical and social factors. Research on the relationship between marital status and TB risk is limited, but social determinants have been recognised as critical factors in TB transmission [90].

Furthermore, those with Qur’anic education had a higher TB prevalence, although this association was not statistically significant after adjustment. Limited access to healthcare information among individuals with less formal education may partially explain this trend. While specific studies on Qur’anic education and TB b risk are scarce, literacy and health knowledge have been linked to TB prevention [22, 91]. As found in the study, individuals categorised as "ART Restart" had a higher TB prevalence and significantly higher odds of developing TB. This finding underscores the importance of continuous access to ART to maintain immune function and reduce TB risk. Interruptions or restarts in ART may weaken the immune system’s ability to control TB infection, leading to higher TB prevalence in this group. Ensuring continuous and uninterrupted access to ART for KP’s living with HIV is crucial in reducing TB co-infection risk. This finding underscores the importance of adherence to ART regimens. Studies have demonstrated that ART initiation and adherence are essential in preventing TB in HIV-positive individuals [92, 93].

In addition, KPs weighing less than or equal to 50 Kg and those with a CD4 count of less than 200 cells/m^3 exhibited higher TB prevalence. However, these associations were not statistically significant in the adjusted ratio. TB risk is closely related to immune and nutritional status, but the lack of significance in this study may be due to sample size limitations. A low CD4 count indicates a compromised immune system, making individuals more susceptible to TB infection. Monitoring CD4 counts and initiating preventive measures, such as isoniazid preventive therapy, for individuals with low counts is crucial to reduce TB risk. Existing literature supports the link between low CD4 counts and increased TB susceptibility [94].

### Limitations of the study

Limitations of this study stem from several factors. Firstly, its sampling approach focused on KPs at specific healthcare facilities, potentially introducing sampling bias and limiting the findings’ generalizability to the broader population. Secondly, the reliance on secondary data from baseline records may result in incomplete or missing information, affecting the overall accuracy and comprehensiveness of the analysis. Thirdly, the cross-sectional design restricts the ability to establish causal relationships and may not capture long-term trends in HIV-TB co-infection. Furthermore, the study’s exclusive focus on six Nigerian states and KPs may not fully represent the country’s diversity, limiting the findings’ applicability to other regions and population groups.

There is a lack of substantial evidence from other research to support the findings of this study, which suggests that both males and females among key populations (KPs) face a similar level of risk for TB (TB) co-infection. The lower TB prevalence among individuals with treatment interruption may not necessarily reflect a true protective effect but could be influenced by factors such as random variation or selection bias within the study sample. Additionally, retrospective studies rely on existing data and medical records, which may not capture all relevant variables or factors that could influence TB risk. The absence of statistically significant differences among age groups in this study might stem from either a limited sample size or a more intricate connection between age and TB within this community. This indicates that age by itself may not be a strong predictor of TB among individuals with HIV in KP.

## Conclusion

This study delves into the complex relationship between TB co-infection and HIV treatment outcomes among Nigeria’s KPs. Notably, it reports a substantial 19.2% TB prevalence among KP’s living with HIV, signalling a significant TB burden within this demographic, aligning with previous findings. The research highlights disparities in TB prevalence across various demographics and clinical factors. While age-related differences emerged, notably those aged 51 and above exhibiting higher TB prevalence, statistical significance was lacking. Nonetheless, it underscores the need for tailored interventions for specific age groups. Regional discrepancies were also apparent, with Jigawa and Cross Rivers states reporting notably high TB prevalence rates, underscoring the importance of region-specific strategies to combat TB co-infection effectively.

Furthermore, the study identifies vulnerable subgroups within KPs, including PWID, divorced individuals, and those with limited formal education, demonstrating higher TB prevalence. This emphasises the necessity for specialised care and support services addressing both medical and social factors impacting these subpopulations. Clinical characteristics like interruptions in ART and low CD4 counts were associated with increased TB prevalence, emphasising the critical need for uninterrupted ART access to strengthen immune function and reduce TB risk. While some associations lacked statistical significance, such as TB prevalence among those with lower body weight or CD4 counts, these factors remain important considerations for TB risk assessment and prevention.

In summary, this study provides valuable insights into the intricate landscape of TB co-infection among KP’s living with HIV in Nigeria, emphasising the need for tailored interventions, region-specific strategies, and specialised care to mitigate TB co-infection risks and improve HIV treatment outcomes. Future research should explore the underlying factors contributing to these disparities and innovative approaches to address this persistent challenge.

## Supporting information

S1 Data (XLSB)
